# ZW and XY Sex Chromosomes Drive Rapid and Distinctive Evolution of Sex‐Biased Gene Expression

**DOI:** 10.1111/mec.70152

**Published:** 2025-10-18

**Authors:** Kevin Hsiung, Sophie Helen Smith, Astrid Böhne

**Affiliations:** ^1^ Leibniz Institute for the Analysis of Biodiversity Change Museum Koenig Bonn Bonn Germany

**Keywords:** cichlid, gene expression, sex chromosome, sexual conflict, sex‐biased gene

## Abstract

Cichlids are a textbook model system of adaptive radiation and a fascinating example of rapid sex chromosome evolution. Yet in these fish, as in most other taxa, the mechanisms causing sex chromosome turnover and the subsequent impact thereof are unknown. Sexual antagonism was long thought to be a driver of sex chromosome emergence, but experimental support remains scarce. Here, we show that sex‐biased genes, often used as indicators of sexual antagonism, are enriched in three different sex chromosome systems of Lake Tanganyika cichlid species that diverged less than 4 million years ago. Moreover, gene expression is feminised in species that transitioned from an XY to a ZW system on the same chromosome. This is achieved by gain of female‐biased genes, increase of female sex‐bias as well as decrease of male‐bias depending on the tissue investigated. We further show that XY sex chromosomes have more male‐biased genes but without higher intensity of sex‐biased expression. A large fraction of sex‐bias in gene expression evolved adaptively, with a stronger signature in females than males. While we find that sex‐bias in gene expression clearly depends on the heterogametic system, we find only weak support for sex‐biased expression priming chromosomes to become sex chromosomes. Overall, we conclude that there is little evidence that sexual antagonism drives sex chromosome emergence but that it likely plays a role during sex chromosome differentiation. We see rapid emergence of antagonistic expression in sex‐linked genes.

## 1 Introduction

The ever‐increasing availability of high‐quality reference genomes has accelerated the discovery of sex chromosomes across the tree of life (Lewin et al. 2018). This growing body of data has initiated a paradigm shift in sex chromosome research (Kratochvíl et al. 2021; Smith et al. 2023), as the evolutionary history of many newly discovered sex chromosomes challenges the longstanding canonical model of sex chromosome emergence and differentiation (El Taher, Ronco, et al. 2021; Furman et al. 2020; Han et al. 2022; Jay et al. 2024; Jeffries et al. 2018; Kuhl et al. 2021; Ma and Rovatsos 2022; Saunders and Muyle 2024). In particular, how the suppression of recombination between the two sex chromosomes is established and extended is a matter of active debate.

Regardless of their evolutionary path, a shared feature of sex chromosomes is their sex‐specific inheritance and main role in defining an organism's sex. Sex chromosome systems are denoted as female heterogametic (ZZ‐ZW), when the female sex has a sex‐specific chromosome (W) and hence produces two types of gametes, and male heterogametic (XX‐XY) when the male sex has a sex‐specific chromosome (Y), resulting in X‐ and Y‐carrying gametes. Note that, while sex chromosomes are the initial decisive agent for an organism to develop as male or female, many other genomic regions can contribute to the actual developmental program of establishing a sex.

Other than sex chromosomes, males and females share a genome. However, the sexes can be evolving towards diverging fitness optima and display dramatically different phenotypes, affecting autosomal and sex chromosomal loci alike. This can result in inter‐sexual conflict on such loci. Resultingly, loci under sexually antagonistic selection (SAS) can simultaneously be beneficial to one sex while detrimental or neutral to the other. Sex chromosomes have been proposed to disproportionately contribute to the resolution of sexual conflict as an advantageous location for genes with sex‐specific fitness effects: specifically, in a male heterogametic XY system where the X spends twice as much time in females than in males and (due to its hemizygous state) being directly exposed to selection in males, the X is predicted to accumulate dominant female‐beneficial alleles as well as recessive male‐beneficial ones (Rice 1984). The reciprocal process is assumed to affect ZW systems, with Z‐linkage facilitating the build‐up of recessive female‐beneficial and dominant male‐beneficial alleles. Additionally, accumulation of male‐beneficial/female‐detrimental genes on the (poorly differentiated) Y can be selectively favored, even if they are not tightly linked to the sex‐determining locus (Rice 1987), as can translocations of genes under SAS from autosomes to the Y (Charlesworth and Charlesworth 1980). However, a key component of resolving sexual conflict is gene expression; in the long term, the genes under SAS could go to fixation on X/Z if gene expression becomes sex‐limited (Rice 1984). The evolution of sex‐limited expression can likewise resolve SAS of autosomal loci.

Directly assessing if a locus is under SAS remains challenging and until now has been mostly done in model systems such as *Drosophila* (Innocenti and Morrow 2010). Sex differences in phenotypes must result from differences in gene expression and thus, sex bias in gene expression is often interpreted as a proxy for SAS (Grath and Parsch 2016). Further, indeed, overrepresentation of sex‐biased genes (SBGs, genes overexpressed in one sex compared to the other) on sex chromosomes (Catalán et al. 2018; Mora et al. 2024) seems to lend support to these predictions concerning SAS. Studies examining SBGs often observe a masculinisation of the Z and feminisation of the X chromosome (Albritton et al. 2014; Allen et al. 2013; Arunkumar et al. 2009; Catalán et al. 2018; Ellegren 2011; Höök et al. 2019, 2024; Mora et al. 2024; Parisi et al. 2003; Song et al. 2021; Storchová and Divina 2006; Zhang et al. 2010) in line with the prediction for selection for dominant female‐beneficial alleles for an XY and dominant male‐beneficial alleles for a ZW system. In the pea aphid and in stalk‐eyed flies, which both have XY systems, a gene expression masculinisation rather than a feminisation of the X has been reported (Baker et al. 2016; Jaquiéry et al. 2013), which could alternatively point to accumulation of recessive male‐beneficial alleles (Rice 1987).

However, the degree to which sex bias in gene expression represents ongoing or rather resolved conflict is debated, and experimental evidence that genes under SAS contribute to the evolution of sex chromosomes by driving the suppression of recombination remains scarce (reviewed in (Smith et al. 2023)). Furthermore, until recently, most studies of sex‐chromosomal gene expression have focused on (highly) differentiated sex chromosomes. Thus, temporal dynamics of sex bias in gene expression on sex chromosomes remain little understood. The degree of differentiation likely contributes to sex bias in gene expression, and we suspect that masculinisation/feminisation of sex chromosomal expression might occur gradually with sex chromosome differentiation, especially on X and Z.

Some systems allow the study of sex chromosomes at different degrees of differentiation. This is, for example, the case in butterflies, where sex‐chromosome‐autosome fusions and large genome rearrangements generated neo‐sex chromosomes multiple times, extending the existing sex chromosome through the addition of autosomal sequence. In the wood white butterfly (*Leptidea sinapis*), which has three neo‐Z chromosomes, the oldest Z chromosome (corresponding to the ancestral lepidopteran Z chromosome fused with a previously autosomal part) harbours more male‐biased genes (MBGs) and fewer female‐biased genes (FBGs) than the two younger Z chromosomes. Contrastingly, the youngest Z chromosome has the highest amount of FBGs, at even greater levels than autosomes (Höök et al. 2024). Similarly, in *Danaini* butterflies, fusion between the existing Z chromosome and an autosome is purportedly driven by the excess of male‐biased genes on this autosome (Mora et al. 2024); indeed, this autosome is the most commonly found autosome involved in neo‐Z fusions in Lepidoptera (Wright et al. 2024). These examples support that SA genes on autosomes drive fusions with sex chromosomes in order to resolve sexual conflict via sex limitation. Consequently, this should further drive sex‐biased expression in a gradual manner.

However, this is not a universal pattern. In the proto‐sex chromosomes of *Rana temporaria* , there is no evidence of sexualisation of gene expression (Ma et al. 2018). Additionally, in the genus *Drosophila*, species with neo‐sex chromosomes had more sex‐biased genes than species without them, and these genes evolved mostly from unbiased genes (Minovic and Nozawa 2024), showing that sex‐biased expression arose after neo‐sex chromosome formation. Taken together, this demonstrates that sex‐biased gene expression is not immediately predictable by the heterogametic system and is intertwined with the evolutionary stage of a sex chromosome. As it stands, we lack conclusive evidence for a causative role of sexual antagonism driving the evolution of sex bias (Dagilis et al. 2022; Patten 2019; Smith et al. 2023).

To further understand the dynamics of sex bias in gene expression of sex chromosomes, comparisons of undifferentiated (young) sex chromosome systems are needed, particularly between species with similar biology that possess both types of heterogametic systems. This would allow studying which type of sex bias evolves under which heterogamety and if sex bias drives sex chromosome emergence or rather results from sex chromosome turnover. In contrast to the conserved, heteromorphic sex chromosomes of groups such as mammals and birds, an overwhelming variety of taxa, including amphibians and teleost fish, have generally younger, diverse sex chromosomes due to frequent sex chromosome turnover events and transitions in heterogametic systems (Devlin and Nagahama 2002; El Taher, Ronco, et al. 2021; Furman et al. 2020; Jeffries et al. 2018).

The East African members of the fish family Cichlidae have an astonishing diversity of sex chromosome systems (Blumer et al. 2025; El Taher, Ronco, et al. 2021; Feller et al. 2021; Ser et al. 2010), which can be attributed to an outstandingly high rate of sex chromosome turnover. Cichlids from the radiation of Lake Tanganyika (comprising about ~250 endemic species (Ronco et al. 2020)) provide an ideal model to study the evolutionary mechanisms of sex chromosome evolution. This system benefits from a well‐resolved phylogenetic reconstruction comprising all species of the radiation (Ronco et al. 2021) and sex chromosomes identified in roughly 30% of the species (El Taher, Ronco, et al. 2021).

**FIGURE 1:**
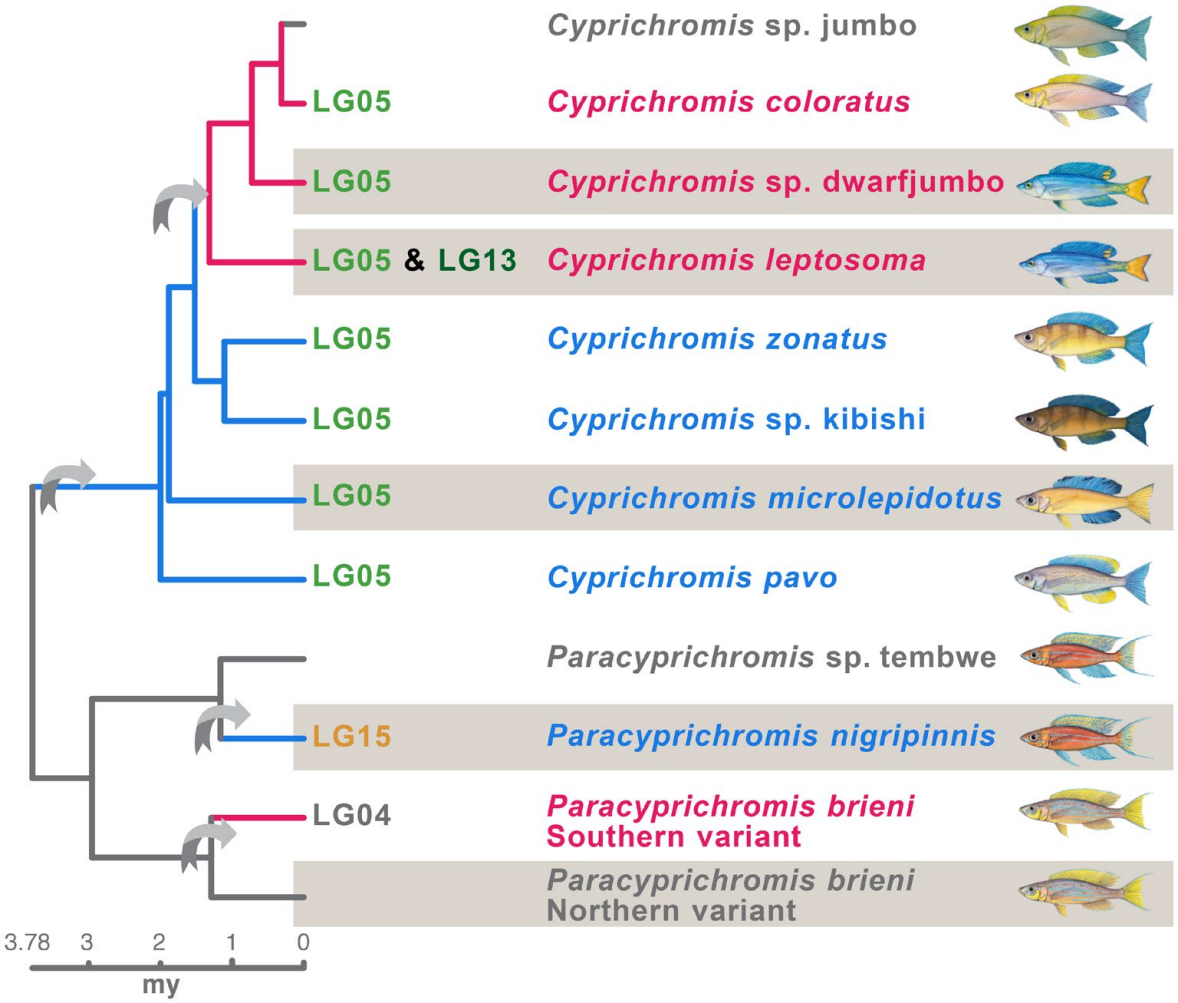
Sex chromosomes in the Lake Tanganyika cichlid tribe Cyprichromini. Sex chromosomes have been described in both genera of the tribe Cyprichromini, *Cyprichromis* and *Paracyprichromis*. The colour of branches and species names illustrates the heterogametic system (female heterogamety ZW red colour, male heterogamety XY blue colour). Chromosomes at tips of branches of the phylogenetic representation indicate sex chromosomes named according to the used reference genome of *Oreochromis niloticus* . Question marks indicate species for which so far, no sex chromosome has been discovered. Arrows indicate sex chromosome turnover events. The five species highlighted with a grey box are investigated in this study. Phylogenetic tree derived from (Ronco et al. 2021) comprising all known Cyprichromini species. Information on sex chromosomes derived from (El Taher, Ronco, et al. 2021) and (Behrens et al. 2022). Fish drawings courtesy of Walter Salzburger. For a distribution of sex‐specific SNPs along the sex chromosomes see Figure S1.

In this study, we investigated one particular Lake Tanganyika lineage, the tribe Cyprichromini, which comprises the genera *Cyprichromis* and *Paracyprichromis* and originated ~4 million years ago (Figure 1). All Cyprichromini are maternal mouthbrooders with a similar torpedo‐shaped body. They display sexual (colour) dimorphism, albeit less pronounced than species of other Lake Tanganyika cichlid lineages. We previously discovered sex chromosomes in eight of the 12 recognised Cyprichromini species, all of which are homomorphic and evolved within this lineage (El Taher, Ronco, et al. 2021) (Figure 1). As we still lack contiguous reference genomes for Cyprichromini, chromosomal assignment of sex chromosomes is according to the linkage groups (LG) of the high‐quality reference genome of an outgroup species, *Oreochromis niloticus* (Nile tilapia). We showed previously that *Paracyprichromis nigripinnis* has an XY sex chromosome system on LG15, with sex‐specific SNPs found along the entire chromosome indicative of wide‐spread reduced recombination (see Supplementary Figure S10 in (El Taher, Ronco, et al. 2021), summarised here in Figure S1). Within the genus *Cyprichromis*, we discovered LG05 as the sex chromosome in 7 out of 8 species. Additionally, LG05 underwent one heterogamety change from XY to ZW. In both the ZW and XY species, sex‐specific SNPs were detected along all of LG05, with a reduced intensity towards the chromosomal end (after 30 Mb). In one of the ZW species, *Cyprichromis leptosoma* , LG05 is fused with LG13, which likewise shows sex‐specific SNPs along most of its length (El Taher, Ronco, et al. 2021). This SNP pattern is indicative of suppressed recombination along most of the sex chromosomal length; however, sex chromosome strata have not been identified so far. The reconstructed ancestral sex chromosome state of Cyprichromini is XY; LG05 likely emerged with the origin of the genus *Cyprichromis* (2–3 Mio years ago). If LG15 is indeed solely a sex chromosome in *P. nigripinnis* , it likely originated ~1 Mio years ago (El Taher, Ronco, et al. 2021). Another study hypothesised that the ZW system on LG05 is older than the XY system (Behrens et al. 2022); however, this is less parsimonious since it is incongruent with the species' evolutionary history and not supported by ancestral state reconstructions for sex chromosome turnovers (El Taher, Ronco, et al. 2021).

Here, we studied gene expression profiles of five Cyprichromini species that capture the major events of sex chromosome turnover in this tribe, to understand the dynamics of sex‐biased gene expression in exchange with sex chromosome evolution (Figure 1). We investigated sex‐specific transcription profiles of somatic tissues (brain, gills, lower pharyngeal jaw, and liver) and gonads for *Cyprichromis* sp. ‘dwarf jumbo’, *Cyprichromis leptosoma* , *Cyprichromis microlepidotus* , *Paracyprichromis nigripinnis* and *Paracyprichromis brieni* (Northern variant) from a previously published dataset (El Taher, Böhne, et al. 2021). Our species selection encompasses the sex chromosome turnover resulting in LG05 as a sex chromosome in the genus *Cyprichromis* (all included *Cyprichromis* species) and LG15 as the sex chromosome in *Paracyprichromis* ( *P. nigripinnis* ) (Figure 1). Within the genus *Cyprichromis*, we further investigated the transition from an XY ( *C. microlepidotus* ) to a ZW system (*C*. sp. ‘dwarf jumbo’, *C. leptosoma* ) on LG05 and the extension of the ZW chromosomes via fusion of LG05 and LG13 ( *C. leptosoma* ). We previously showed that sex‐linkage seems to span most of the species' sex chromosomes (note that the reduced number of sex‐specific SNPs was mostly detected in regions with overall low SNP density, see Supplementary Figure S10 of El Taher, Ronco, et al. 2021; Ronco, et al. 2021). However, the sex chromosomes show no signs of extensive sequence degeneration supported further by similar sequence coverage levels in both sexes. We also found no evidence for gene loss from the Y/W chromosomes.

Now, this opportune dataset allows us to investigate gene expression in both types of heterogametic systems involving different sex‐linked chromosomes, balanced by a stable background of closely related species with common life‐history traits and homomorphic sex chromosomes. We applied measures of differential gene expression analysis to study the evolution of sex bias in gene expression in soma and gonad tissue in the above‐described species set and assessed the amount and strength of male‐ and female‐biased genes in a comparative species framework. We further tested if sex bias in gene expression evolved selectively. We hypothesised that sex chromosomes differ in their sex‐specific expression compared to autosomes and predicted that sex bias in gene expression on the here studied sex chromosomes is distinct from expression patterns of heteromorphic sex chromosomes.

## 2 Materials and Methods

### 2.1 Sequencing Data

The sequencing data used in this study were generated in a previous study (El Taher, Böhne, et al. 2021). We obtained these Illumina strand‐specific single‐end transcriptome reads (read length 125 bp) from GenBank under the BioProject ID PRJNA552202. We retrieved all data available for Cyprichromini, i.e., *C*. sp. ‘dwarf jumbo’, *C. leptosoma* , *C. microlepidotus* , *P. brieni* (Northern variant) and *P. nigripinnis* . Typically, three male and three female replicates (details in Table S1) of each species were sequenced for five tissues (gonads, brain, gills, liver, and lower pharyngeal jaw). For *C. leptosoma* , no data were available for liver and lower pharyngeal jaw.

### 2.2 Quality Filtering, Mapping and Read Counting

Raw reads were quality‐filtered with Trimmomatic ‘0.39’ (Bolger et al. 2014) with a sliding window size of 4 bp, a minimum window quality of 15 and a minimum read length of 80 bp (i.e., ⅔ of the initial read length). Adapter removal was also performed with Trimmomatic ‘0.39’. The quality of reads was assessed before and after trimming using FastQC ‘0.11.9’ and MultiQC ‘1.12’ (Ewels et al. 2016). Due to the lack of a well‐assembled and annotated reference genome for Cyprichromini and to allow comparisons to previous studies on cichlids of Lake Tanganyika, reads were mapped against the chromosome‐scale genome assembly of the Nile tilapia ( *O. niloticus* , RefSeq assembly version GCF_001858045.2 (BioProject: PRJNA344471), mapping statistics detailed in Table S2), a closely related species that is equidistant to all focal species of this study. This reference is well annotated, and previous less contiguous assembly versions of it already allowed producing reliable mapping rates for Lake Tanganyika cichlid read data (El Taher, Böhne, et al. 2021; Lichilín et al. 2021). The Nile tilapia has a diploid chromosome number of 2n = 44, a common karyotype among African cichlids (Ozouf‐Costaz et al. 2017). To the best of our knowledge, no karyotype information is available for members of the tribe Cyprichromini.

After genome indexing, reads were mapped to the reference genome with STAR ‘2.7.10a’ (Dobin et al. 2013) using two‐step mapping. Splice junction information was generated in an initial first‐pass mapping step for all individual samples; a second 2‐pass mapping step then used first‐pass splice junctions across all samples to remove false positive junctions. First‐pass mapping settings were as follows: ‘STAR ‐‐runMode alignReads ‐‐outFilterMultimapNmax 1 ‐‐runThreadN 4’, and 2‐pass mapping settings were ‘STAR ‐‐runMode alignReads ‐‐sjdbFileChrStartEnd SJFILES ‐‐limitSjdbInsertNsj 2000000 ‐‐runThreadN 4’. Alignments in BAM format were assigned to genes and counted using HTSeq ‘2.0.1’ (Anders et al. 2015) with settings ‘htseq‐count ‐f bam ‐r pos ‐m union ‐s reverse ‐t gene’. Samtools ‘1.10’ (Li et al. 2009) was used to sort and create indexes for BAM files of aligned reads. The total number of input reads and mapped reads is detailed in Table S2.

### 2.3 Differential Gene Expression Analysis

All count data were processed and analysed with R ‘4.0.3’ using the DESeq2 library ‘1.30.1’ (Love et al. 2014) and R package ggplot ‘3.4.2’ (Wickham 2016). Following the DESeq2 recommendations, we retained only genes with a minimum read count of at least 5 in more than three samples for differential gene expression analysis. After this removal, we obtained final datasets with > 25,000 genes for each species (details in Table S3). Count data were normalised through DESeq2's internal medium of means normalisation method. Significant effect of sex on gene expression was determined by fitting count data per species of each tissue through a generalised linear model (GLM) with a negative binomial distribution as integrated in DESeq2. DESeq objects were created (i) per species using all organs with a model using count data of all tissues with sex and tissue as explanatory variables, (ii) per species for each tissue separately with a model with just sex as the explanatory variable. *p*‐values were attained through a Wald test and corrected for multiple testing with the Benjamini & Hochberg method (Benjamini and Hochberg 1995) with a false discovery rate (FDR) of < 0.05 as implemented in DESeq2. As we expect ongoing sexually antagonistic selection to result in rather nuanced differences in sex expression (as discussed in Cheng and Kirkpatrick 2016; Innocenti and Morrow 2010; Sayadi et al. 2019; Wright et al. 2018), we did not impose a Log_2_ fold‐change (LFC) cut‐off when defining significantly sex‐biased genes but solely relied on significance levels. Therefore, genes with a resulting adjusted *p*‐values (*p*‐adj) < 0.05 were considered indicative of significantly sex‐biased genes. All other expressed genes were considered unbiased. Gene locations on chromosomes were derived from the reference genome annotation.

Principal component analysis (PCA) was performed to examine expression patterns within species and for all species combined. For this, counts were first transformed with the mean of variance stabilising transformation (VST) as implemented in DESeq2. No outlier samples were identified and removed, which was expected as the dataset in this study included only all samples/libraries that had been previously validated in (El Taher, Böhne, et al. 2021). To test for a difference in sex bias of SBGs on sex chromosomes and autosomes, we applied a Wilcoxon‐Mann–Whitney‐Test for pairwise comparisons and a Kruskal–Wallis test for comparisons involving three groups.

Sex chromosomes and heterogamety systems for *C*. sp. ‘dwarf jumbo’, *C. leptosoma* , *C. microlepidotus* and *P. nigripinnis* were previously identified by (El Taher, Ronco, et al. 2021), while the identity of the sex chromosome and heterogametic sex for *P. brieni* remain unknown. *P. brieni* was thus excluded in comparisons of SBGs across sex chromosomes and autosomes. For comparisons of genes on sex chromosomes versus those on autosomes, we excluded mitochondrial genes and genes that are located on unplaced scaffolds in the reference genome. When comparing overall expression intensity between autosomes and sex chromosomes, TPM (transcripts per kilobase million) normalised count values were used.

### 2.4 Analysis of Gene Expression Evolution and Directional Selection

To investigate whether SBG expression has evolved adaptively, we used an existing model (Hsieh et al. 2003; Moghadam et al. 2012; Rifkin et al. 2003; Scharmann et al. 2021; Zemp et al. 2016), which compares divergence in expression within a focal species to another related species with respect to variation within the focal species' replicates.

The model assumes that genes under directional selection simultaneously show an increase in between‐group expression and a decrease in within‐group variation (low expression variance between replicates). The measure of divergence in expression, ∆*x*, is calculated for each gene using the formula ∆*x* = *d*/*fr*, where d (divergence) is calculated as *d* = (mean relative gene expression of focal species) – (mean relative gene expression of related species)/(mean relative gene expression of focal species); *r* (range) is calculated as *r* = ((largest expression value of focal species) – (lowest expression value of focal species))/(mean expression value of focal species). To correct for differences in sample sizes, *r* is multiplied by *f*, where *f* = squareroot(*mN* – 1/*mN* – *m*), *m* is the sample size, and *N* (3) is the most common sample size. We calculated ∆*x* values for each gene using the log2 transformed TPM normalised count values for each *Cyprichromis* species with respect to the outgroup *P. brieni* representing the earlier branching genus *Paracyprichromis* within the Cyprichromini species tree (Figure 1). Genes with an absolute ∆*x* value greater than 1 are considered to be under directional selection. ∆*x* values greater than 1 indicate upregulation in expression, and ∆*x* values below −1 represent downregulation. When calculating ∆*x* values for *P. brieni* , *P. nigripinnis* was used for comparison. Fisher's exact tests were conducted to test if gene categories differed in terms of putative directional selection.

### 2.5 Rank–Rank Hypergeometric Overlap Test

To further identify potential concordance and disconcordance in sex bias of gene expression between species, we performed threshold free rank–rank hypergeometric overlap (RRHO) analyses (Plaisier et al. 2010). In each pairwise comparison, we included only genes where expression was detected in both species. Using the adjusted *p*‐values obtained from the above described DESeq2 analysis of each species, the degree of differential expression (DDE) for each gene was calculated as –log10(*p*‐value)*effect size direction as recommended in Plaisier et al. (2010), with negative values indicating overexpression in females and positive values overexpression in males. Using the R package RRHO ‘1.44.0’ (Plaisier et al. 2010), DDE values were ranked and binned using the package's own default stepsize calculation (i.e., squareroot (genes analysed), rounded up to the next integer). Multiple testing correction was performed using the Benjamini and Yekutieli method (Benjamini and Yekutieli 2001).

## 3 Results

To investigate the impact of sex chromosome turnover and heterogamety transition on gene expression in young, homomorphic sex chromosome systems, we (i) analysed global gene expression differences between the sexes (ii) compared tissue‐specific patterns and (iii) disentangled selective forces shaping gene expression.

### 3.1 Tissue Type Shapes Global Expression Profiles Similarly in Closely Related Species

**FIGURE 2:**
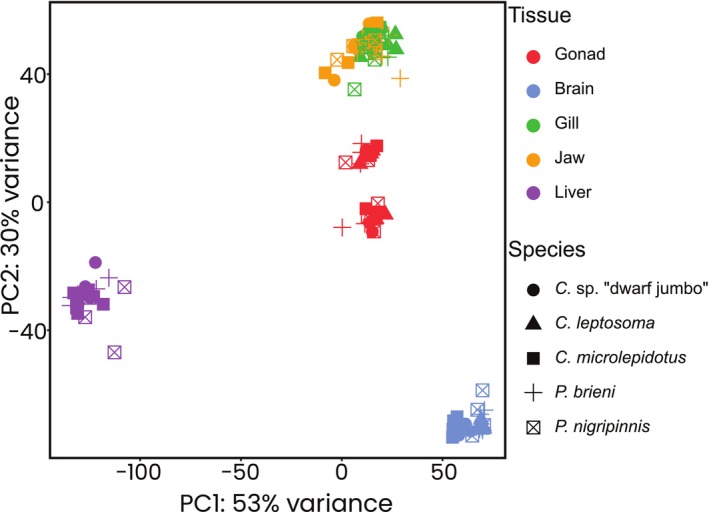
Global gene expression. Global gene expression profile of all investigated samples is visualised with a principal component analysis (PCA), which across species separates tissue types and within the gonads additionally male and female samples. Proportions of variance explained by the first two principal components (PC) are indicated on the x‐ and y‐axis, respectively. Samples are coloured according to organ type and shapes denote species as explained in the inset.

A global gene expression principal component analysis (PCA) revealed similar tissue‐specific expression profiles amongst species, with the exception of gills and lower pharyngeal jaw (Figures 2 and S2). These two bony tissues had overlapping expression patterns, which is unsurprising as the lower pharyngeal jaw is derived from the fusion of the left and right fifth ceratobranchials and is developmentally related to the gills (El Taher, Böhne, et al. 2021; Liem 1973). The effect of sex on gene expression was clearly visible in gonads, supporting ovaries and testes as distinct sex‐specific organs. Uniquely in *C. microlepidotus* , sex also noticeably affected liver gene expression, separating male and female replicates (Figure S2).

### 3.2 Globally Sex‐Biased Genes Are Overrepresented on Sex Chromosomes

**FIGURE 3:**
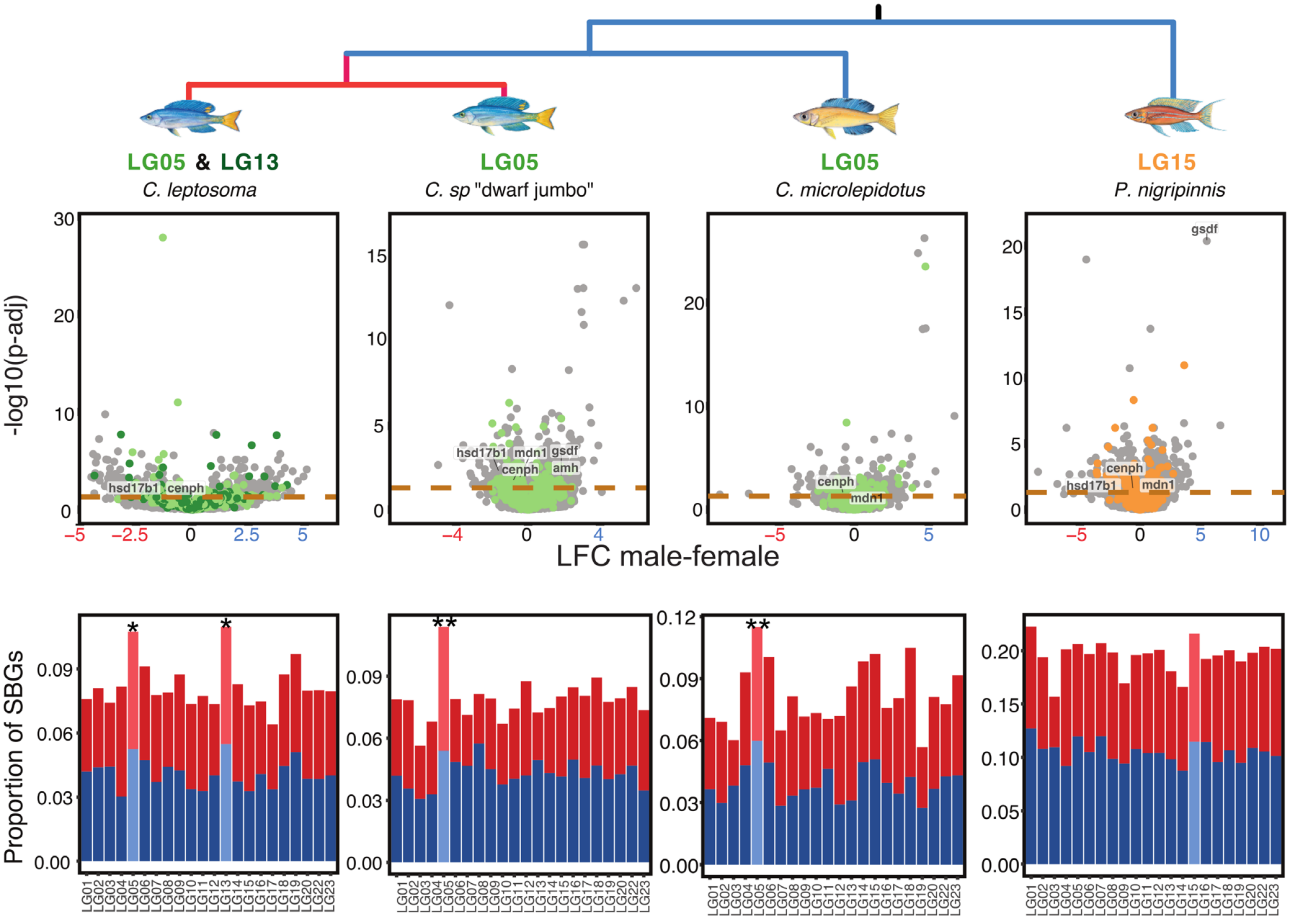
Sex‐biased gene expression profiles per species. Panels in the upper row show volcano plots of sex difference in gene expression of all genes across all tissues per species. Dotted lines correspond to an adjusted *p*‐value of 0.05. Genes above the dotted lines are considered sex‐biased. Coloured dots represent genes located on the respective sex chromosome (light green: LG05, dark green: LG13, orange: LG15). LFC = log2‐fold change in gene expression; negative values indicate female bias, positive values male bias. LG = linkage group. Panels in the lower row show the distribution of sex‐biased genes (SBG) across the reference genome, with female‐biased genes in red and male‐biased genes in blue colour. Sex chromosomes of respective species are represented by bars of brighter colouration. * = *p*‐value < 0.05; ** = *p*‐value < 0.01 of Fisher's exact test.

We next investigated gene expression with two approaches. First, we aimed to analyse global effects of sex on gene expression. For this, we combined all tissues into one analysis, controlling for tissue effect and thus maximising the effect of sex, hereafter referred to as the tissue‐combined model (see Methods for details). With this approach, the *Cyprichromis* species showed similar proportions of ~8% SBGs (*C*. sp. ‘dwarf jumbo’ 1932/7.75%, *C. leptosoma* 2007/7.91% and *C. microlepidotus* 2014/7.83%). *P. nigripinnis* showed the highest amount of SBGs (4937/18.8%) and *P. brieni* the lowest amount (147/0.57%) (Table S3). The SBGs included several genes with known functions in sexual development: *Amh* (*anti‐müllerian hormone*) and *gsdf* (*gonadal soma‐derived factor*) were male‐biased and the most significantly sex‐biased gene in *P. brieni* and *P. nigripinnis* , respectively (Figure 3, Table S4), consistent with their known functions in male sexual development (Du et al. 2022; Edvardsen et al. 2022; Holborn et al. 2022; Liu et al. 2022; Sánchez‐Baizán et al. 2024; Smith et al. 2023; Yu et al. 2024; Zhou et al. 2019).

The female‐specific sex determiner *hsd17b1*, (Fan et al. 2021; Koyama et al. 2019) had female‐biased expression in *C*. sp. *‘dwarf jumbo’, C. leptosoma* and *P. nigripinnis* .

Sex chromosomes had the highest proportion of SBGs in all *Cyprichromis* species (Figure 3) and were resultantly significantly enriched in SBGs (Fisher's exact test, *C*. sp. ‘dwarf jumbo’ *p* = 1.49e‐05*; *C. leptosoma* : LG05 *p* = 0.0019*, LG13 *p* = 0.0044*; *C. microlepidotus: p* = 7.23e‐05*; Table S5). In *P. nigripinnis* , the sex chromosome LG15 showed the second highest proportion of SBGs (the highest being LG01).

In ZW *C*. sp. ‘dwarf jumbo’, the increase in SBGs on sex chromosomes was driven to a greater extent by FBGs than MBGs (proportion of FBGs on LG05 was greater than on any other chromosome, Table S6). In the ZW *C. leptosoma* , the enrichment of SBGs was driven by both MBGs and FBGs: here LG13 and LG05 exhibited the highest proportion of FBGs and MBGs across all chromosomes. Both ZW species had slightly but not significantly more FBGs than MBGs on LG05 (Figure 3; *C*. sp. ‘dwarf jumbo’ LG05 F:M = 71:64; *C. leptosoma* LG05 F:M = 65:62 and LG13 F:M = 47:47). In the LG05‐XY *C. microlepidotus* , SBG enrichment on LG05 was driven by MBGs. Both XY species had more MBGs than FBGs on their respective sex chromosomes (Figure 3; *C. microlepidotus* LG05 F:M = 65:71; *P. nigripinnis* LG15 F:M = 111:126, difference not significant).

We also found the highest proportion of FBGs in *P. brieni* (no sex chromosome known) on LG15, although it is seemingly not a sex chromosome in this species (Table S3, (El Taher, Ronco, et al. 2021)). Likewise, the second highest proportion of MBGs and FBGs of *C. microlepidotus* was on LG15 and LG13, respectively.

Generally, we found species to share few SBGs with each other based on comparing the per‐species derived SBGs (Table S7 and Figure S3). To complement this analysis, we decided to further investigate potential concordance in sex‐biased expression patterns across shared expressed genes using rank–rank hypergeometric overlap (RRHO) analysis. RRHO enables a threshold‐free analysis of ranked genes, potentially detecting more subtle concordant expression patterns. In line with the limited amount of shared SBGs, we found medium levels of concordant sex‐biased expression (pairwise spearman's *ρ* 0.585–0.713), with 52.3%–75.3% of genes tested showing concordant sex‐biased expression amongst species (Table S8). All FBGs and MBGs identified by DESeq2 as shared between two species were always concordantly expressed according to RRHO. We did not detect a consistent chromosome wide difference of concordant sex‐biased expression between autosomes and sex chromosomes, nor between ZW and XY species across the species comparisons based on RRHO. Comparing concordant sex‐biased expression on LG05, the three species that possess LG05 as a sex chromosome showed on average 63.2% of tested genes to be concordantly sex‐biased, while comparisons with *P. nigripinnis* , where LG05 is an autosome, showed on average 61.5% of tested genes to be concordantly expressed. Further examining LG05 between XY‐LG05 to ZW‐LG05 comparisons likewise did not show differences in concordant expression. Here the average proportion of concordantly expressed genes was 64.0% when comparing *C. microlepidotus* (XY‐LG05) to *C*. sp. ‘dwarf jumbo’ (ZW‐LG05) or *C. leptosoma* (ZW‐LG05) and 61.6% between *C*. sp. ‘dwarf jumbo’ and *C. leptosoma*.

As RRHO did not reveal patterns of chromosome or genome wide differences between species, we next focused on the overlap of significant SBGs out of the DESeq2 analyses across the three LG05 sex chromosome species. We found that the two ZW species shared significantly more FBGs on the sex chromosome LG05 than on autosomes (Fisher's exact test; *p* = 0.0014*, Table S7). In the ZW *C*. sp. ‘dwarf jumbo’, the most significantly sex‐biased gene on LG05 was an FBG, *ccndbp1*, and also one of the shared FBGs with *C. leptosoma* (unbiased in the two XY species). In *C. leptosoma* , the two most significantly sex‐biased genes were both FBGs on LG05, LOC100693948 (ATP‐dependent RNA helicase DDX19A) and *acad9* (acyl‐CoA dehydrogenase family member 9), the latter was also female‐biased in *C*. sp. ‘dwarf jumbo’ and again unbiased in the XY species. *Ddx19a* is ovary‐biased in the Nile tilapia and acts in the immune system, which typically differs between males and females (Campbell et al. 2021).

To gain insight on whether the similar expression of LG05 FBGs in the two ZW species was indeed an effect of their shared sex chromosome and novel heterogametic system as opposed to being purely due to the shorter time of species divergence between *C*. sp. ‘dwarf jumbo’ and *C. leptosoma* , we tested for shared SBGs on LG13. *C*. sp. ‘dwarf jumbo’ and *C. leptosoma* were less likely to share FBGs on LG13 than on autosomes (*p* = 0.039*). We detected no significant associations in pairwise analysis for MBGs (Table S7).

All *Cyprichromis* species shared two FBGs (*mdm4*, an apoptosis regulator and LOC100534483, a glutathione peroxidase) and two MBGs (*abhd6* and uncharacterised gene LOC106098860) on LG05, (Figure S3, Table S7).

In *C. leptosoma* , amongst the 10 most significant SBGs, four were on LG13. Three of these genes have a function in sexual development: the FBG *mad2l1bp* in spermatogenesis (Blázquez et al. 2017); the MBG *cyp17* is important in sex determination and gonadal development in many teleosts, and when disrupted causes sex reversal (Meng et al. 2019; Yang et al. 2021; Zhai et al. 2022), and the MBG *lhcgr* (MBG), luteinizing hormone/choriogonadotropin receptor, is necessary for steroid hormone production, gonad development and reproductive processes (Narayan 2015).

Meanwhile in the LG15 species *P. nigripinnis* , the most significantly supported SBG is an MBG on LG15 and is also the most sex‐biased gene, *lrfn5b*, which regulates synaptic development (Lie et al. 2018).

### 3.3 XY and ZW Species Evolve Global Sex Bias in Gene Expression Differently

**FIGURE 4:**
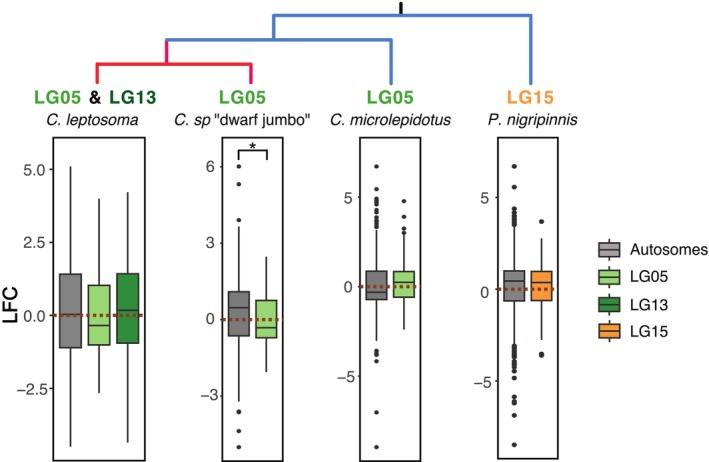
Distribution of male–female expression difference between autosomes and sex chromosomes. Boxplots depict the distribution of change in gene expression in sex‐biased genes (SBGs) across chromosome groups. Dotted lines correspond to a log2‐fold change (LFC) of zero. The box plot centre lines represent the median, the box limits represent the upper and lower quartiles, and the whiskers represent 1.5× the interquartile range. LG = linkage group, negative values indicate overexpression in females, positive values overexpression in males. *C*. sp. ‘dwarf jumbo’ *p* = 0.01018*; *C. leptosoma* *p* = 0.65; *C. microlepidotus* *p* = 0.2644; *P. nigripinnis* *p* = 0.7794.

We next investigated sex‐bias intensity of gene expression across chromosomes in the tissue‐combined model. Considering all expressed genes, averaged expression was male‐biased for all chromosomes of all species (Figure S4). Yet, compared to autosomes, the median LFC of SBGs on the ZW chromosomes was female‐biased, whereas on XY chromosomes it was male‐biased (Figure 4). The younger sex chromosomal region LG13 of *C. leptosoma* was on average male‐biased.

**FIGURE 5:**
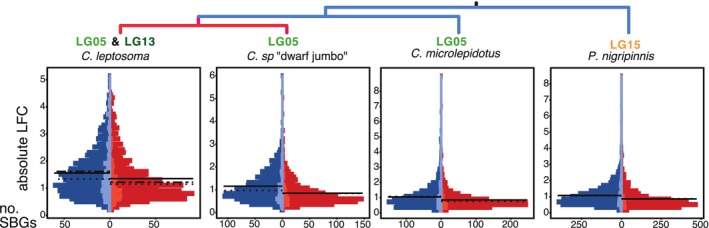
Distribution of male–female expression depending on sex bias. Histograms show the distribution of absolute log2‐fold change in gene expression (LFC) values of sex‐biased genes (SBGs) for the tissue‐combined model per species. Female‐biased genes (FBGs) are shown in red colour and male‐biased genes (MBGs) in blue. Sex chromosomal SBGs are depicted in lighter colour towards the centre of each graph, autosomes are depicted with more saturated red/blue. Solid lines across each side of the graph represent the autosomal mean LFC, dotted lines represent the sex chromosomal mean. For *C. leptosoma* , dotted lines correspond to LG05, dash‐dotted lines represent LG13.

The shift to averaged female‐biased expression on the ZW chromosome pattern was not driven by an increase of LFC of FBGs alone but also by an increase in the number of FBGs (Figure 5). Overall, MBGs on autosomes and sex chromosomes showed stronger bias than FBGs. Still, in the ZW species, the overall difference in LFC between FBGs and MBGs was lower on LG05 than on autosomes (Figure 5). In ZW *C*. sp. ‘dwarf jumbo’, we observed lower LFC of MBGs on LG05 compared to autosomal MBGs. In ZW *C. leptosoma* , LFC on sex chromosomes was lower for both FBGs and MBGs compared to autosomes, with a stronger reduction in MBGs (Figure 5). In contrast, in the two XY species, the average LFC for FBGs and MBGs did not differ between autosomes and sex chromosomes.

We did not detect a clear positional effect of sex‐biased gene expression along the sex chromosomes (Figure S5), suggesting that the observed gene expression trends in sex bias are not limited to a specific region on the sex chromosomes.

### 3.4 ZW Sex Chromosomes Are Feminised and XY Sex Chromosomes Masculinised but With Different Mechanisms

We next investigated sex‐biased expression within each tissue separately (i.e., tissue‐specific model) and unsurprisingly found gonads to have the largest amount of SBGs, i.e., 64.01%–76.76% of all expressed genes and more MBGs than FBGs (Table S3). The somatic organs showed a much lower proportion of SBGs (0.0097%–2.95% of expressed genes; Table S3). Of these, the liver had the largest number of SBGs.

**FIGURE 6:**
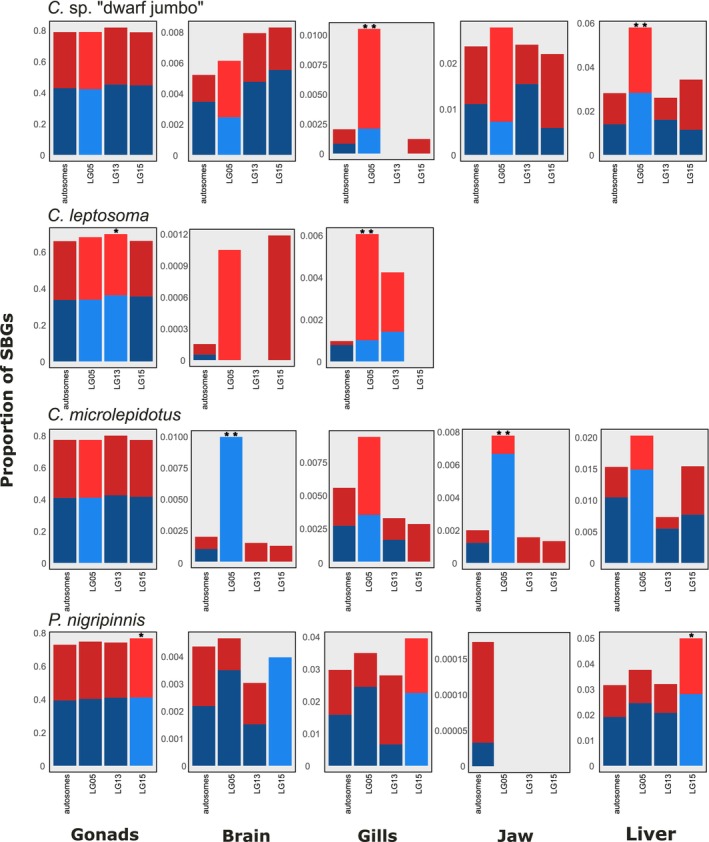
Distribution of SBGs in each tissue. Barplots show the proportion of male‐biased genes (MBGs, blue) and female‐biased genes (FBGs, red) out of all expressed genes in each tissue per species. Respective sex chromosomes of each species are more brightly coloured. * = *p*‐value < 0.05; ** = *p*‐value < 0.01 of Fisher's exact test.

On the sex chromosomes, we detected more MBGs than FBGs in the gonads, except in the ZW species *C. leptosoma* which had more FBGs than MBGs on LG05, but not on the younger LG13 segment (Table S3, Figure 6). In the somatic tissues, however, ZW species overall showed more FBGs on LG05 than on autosomes, while in the XY species, MBGs were more abundant on the respective sex chromosomes (with the exception of the gill tissue in *C. microlepidotus* ), aligning with our observation from the tissue‐combined model (Figures 3 and 6). There was no unambiguous effect of sex chromosomal location and heterogametic system on gonad gene expression (Figure 6). Hence, these sex‐specific organs are not the major drivers of the global effect of the heterogametic system on gene expression described above.

As in the above‐described global tissue‐combined model, we found little overlap in SBGs across species when somatic tissues were analysed separately (Figure S6). In the tissue‐specific models, shared MBGs and FBGs did not appear more frequently on LG05 than on autosomes (Figure S6 and Table S7).

### 3.5 Tissue‐Specific Expression Profiles Are Feminised in ZW Species and Masculinised in XY Species

Based on the tissue‐specific models, across all genes and tissues, sex chromosomes showed similar distributions of male–female gene expression ratios as autosomes (Figure S7), indicating equal expression of the sex chromosome pair compared to autosomes.

**FIGURE 7:**
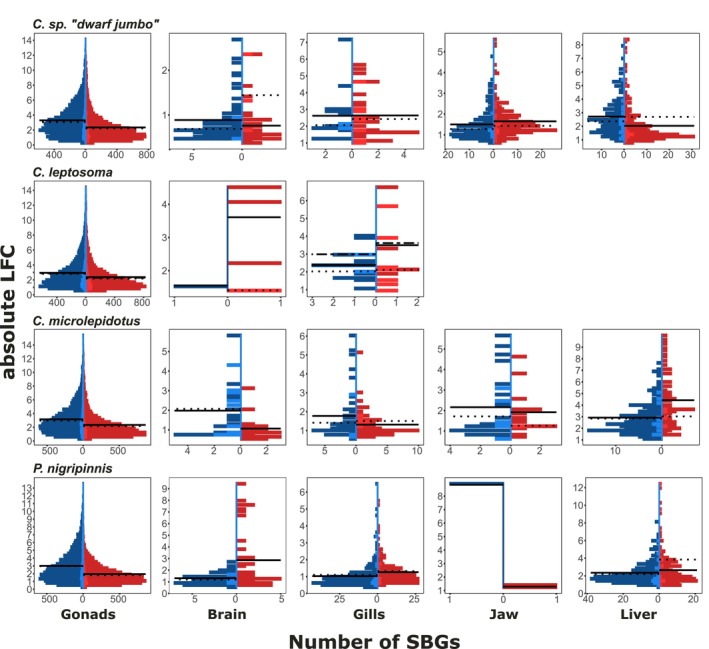
Distribution of male–female expression depending on sex bias. Histograms show the distribution of absolute log2‐fold change in gene expression (LFC) values of sex‐biased genes (SBGs) for each tissue per species. Female‐biased genes (FBGs) are shown in red colour and male‐biased genes (MBGs) in blue. Sex chromosomal SBGs are depicted in lighter coloured shading towards the centre of each graph, autosomes are depicted with more saturated red/blue. Solid lines across each side of the graph represent the autosomal mean LFC, dotted lines represent the sex chromosomal mean. For *C. leptosoma* , dotted lines correspond to LG05, dash‐dotted lines represent LG13.

In addition to their greater number, MBGs in the gonads had higher LFC values, i.e., stronger bias than FBGs across all species on sex chromosomes and autosomes, irrespective of heterogamety system (Figure 7). In contrast, in the soma, sex chromosomal FBGs showed stronger bias than MBGs in the ZW species, while in the XY species there was no clear pattern (Figure 7). This was even found to a greater degree on LG13 in *C. leptosoma*, which has been sex‐linked for an even shorter time than LG05. Note that we did not detect this pattern with the tissue‐combined model where we instead had evidence for feminisation from an increase in FBG number compared to MBGs. We did not find the reciprocal pattern in XY species; while the (older) sex chromosomes of XY species were generally masculinised in proportion to SBGs, we did not find MBGs to have a stronger LFC than FBGs, nor did sex chromosomal SBGs show higher LFC than autosomal ones.

We next investigated genes that evolved sex‐biased expression with sex chromosome emergence. In the soma, SBGs on sex chromosomes in each species mainly evolved from unbiased genes, with some change in the direction of sex bias between species (Figure S8). The most strongly sex‐biased genes were generally female‐biased in *C*. sp. ‘dwarf jumbo’ and *C. leptosoma* , but male‐biased in *C. microlepidotus* , and commonly located on the sex chromosome LG05 (Figure S8).

### 3.6 Potentially Stronger Selection on Female Gene Expression

As opposed to resulting from directional selection and being adaptive, it is also possible that gene expression changes result from drift, i.e., the emergence of sex bias in expression is due to neutral or random processes. While the absolute number of SBGs in somatic tissue was rather low, many of those that we detected had strong statistical support and high LFC values, which supports the idea that these changes are adaptive and cast doubt on drift as their evolutionary driver (Figure S8).

To address this, we tested for signals of directional selection on gene expression for SBGs in males and females by accessing the divergence in expression captured by the ∆x ratio. In ovaries and testes, between 28.25%–42.41% and 26.81%–39.04% of SBGs were under directional selection, respectively (Table S9). This signal was even stronger in the soma, comprising 27.37%–100% of SBGs (excluding the gills of *P. brieni* , where no MBGs were found, and the sole FBG did not show signals of directional selection).

Within most somatic tissues, more SBGs were under putative directional selection in females than in males. This was not the case only in the liver and gills of *C. microlepidotus* , gills of *C. leptosoma* and jaw, brain and gills of *P. brieni* , where either more SBGs in males were under putative directional selection or it was equal between males and females (Table S9). SBGs were more likely to be under directional selection than unbiased genes in the gonads of all species except *P. brieni* (Table S10). In somatic tissue, we found a similar pattern (Table S10). SBGs on sex chromosomes were not more likely to be under putative directional selection compared to those on autosomes (Table S11).

## 4 Discussion

The role of sex‐biased gene expression and sexually antagonistic selection in sex chromosome turnover has been widely debated, alongside recent advances in sequencing technology, transcriptomic methods and investigations of novel sex chromosomes. We explore the dynamics of SBGs throughout species divergence in five closely related African cichlid species of the tribe Cyprichromini and reveal how gene expression is coupled to turnovers of the sex chromosome and changes in the heterogamety type. We find enrichment of sex‐biased expression through both gain of SBGs and increased strength of sex bias to evolve rapidly with sex chromosome turnover. We propose that sex bias mostly evolves in response to sex chromosome turnovers and especially heterogamety change, rather than being a prerequisite for turnover events.

Based on a global gene expression analysis, we detected a moderate level of concordant sex bias across the genome using RRHO analysis. General correlations of expression have been previously shown to be much higher within Lake Tanganyika cichlids (El Taher, Ronco, et al. 2021), suggesting potential for faster evolution of sex‐biased expression than expression in general. When focusing on statistically supported sex‐biased expression (i.e., DESeq2), we find all sex chromosomes in the genus *Cyprichromis* to be enriched in SBGs, including the most recently fused LG13 in *C. leptosoma* . We see more enrichment of MBGs in the XY systems and FBGs in the ZW species respectively, although these differences are subtle. Still, this enrichment of SBGs on sex chromosomes may align with predictions about the effect of sexual conflict and its resolution through the evolution of (strong) sex‐biased gene expression (Ellegren and Parsch 2007). Given the young age of the sex chromosomes within our system, and the even younger fusion of LG13 with LG05 in *C. leptosoma* , our results suggest that sex bias in gene expression on sex chromosomes can evolve quickly.

Gene expression on Z chromosomes is often masculinised and feminised on X chromosomes (Albritton et al. 2014; Allen et al. 2013; Arunkumar et al. 2009; Catalán et al. 2018; Ellegren 2011; Höök et al. 2019, 2024; Mora et al. 2024; Parisi et al. 2003; Song et al. 2021; Storchová and Divina 2006; Zhang et al. 2010). However, this pattern is often explained by a dosage effect. Recombination suppression over time leads to gene loss on the Y/W chromosome, creating chromosomal dosage differences between sexes which in turn can have an effect on gene expression levels (Mank 2013). Thus, the patterns we observe could represent sex‐specific expression from X or Z under hemizygosity, potentially driven by recessive female‐ and male‐beneficial genes on the Z and X, respectively. However, given the low degree of sex chromosome differentiation in our target species (El Taher, Ronco, et al. 2021), we have no indications for hemizygosity of genes on the sex chromosomes. Here tested, we did not see an effect of dosage compared to autosomal expression levels. We hence propose that, within Cyprichromini, the higher proportion of FBGs compared to MBGs on the sex chromosome in female heterogametic species, and *vice versa* for male heterogametic species, stems from novel expression gained on the respective sex‐limited chromosomes (W and Y) indicating a sex‐specific role for these genes. Simultaneously, we observed a reduction of male‐bias intensity of MBGs in the ZW species. We speculate that this might be attributed to the lost expression from the Y chromosome, which could suggest that the ZW system evolved from the X chromosome.

While the differences in sex‐biased expression between the XY and ZW systems are subtle, they are significant and derived from the analysis of several tissues, increasing statistical support and reducing noise. This would suggest that especially SBGs found from the tissue‐combined model are genes under either ongoing or resolved SAS through their novel location on a sex chromosome. This finds mixed support across species: Studies of other systems with homomorphic sex chromosomes, yet older ones than those of cichlids, did not find enrichment of SBGs on sex chromosomes (e.g., in spiny frogs (Xiao et al. 2025), common frogs (Ma et al. 2018), scallops (Han et al. 2022), threespine stickleback (Sylvestre et al. 2025)) or opposing patterns to what we observed (overrepresentation of male‐biased genes in the nonrecombining region on young ZW chromosomes in the plant *Amborella trichopoda* (Käfer et al. 2022)). Still, in beetles, where ancestral X chromosomes and older neo‐X chromosomes are higher expressed in ovaries than in testes, this difference is not detectable in a young neo‐X chromosome, also suggesting that sex‐biased gene expression did not prime these sex chromosomes to emerge in the first place but that sex‐specific gene expression takes time to evolve (Bracewell et al. 2024).

In *Drosophila albomicans*, *americana* and *miranda*, adult MBGs are overrepresented on the ancestral X chromosome, whereas the neo‐sex chromosomes in these species are enriched in larval FBGs and MBGs. Similar to our observations, in these species, many of the genes acquired sex‐biased expression after linkage to the neo‐sex chromosome (Minovic and Nozawa 2024). Also matching our observations, neo‐W chromosome genes in the Crested Ibis evolved ovary‐biased gene expression from unbiased expression in the gonad (Xu et al. 2024).

In contrast to LG05 where SBGs mostly evolved from unbiased genes, we find LG15 and LG13 to possess high proportions of FBGs and MBGs in *P. brieni* and *C. microlepidotus* where they are not sex chromosomes. This suggests that LG13 and LG15 in cichlids in general harbour genes under SAS or related to sex‐specific functions, priming them for recruitment as sex chromosomes in Cyprichromini. The neo‐sex chromosome LG13 in *C. leptosoma* contains *mad2l1bp* and *lhcgr* which function in spermatogenesis and steroid hormone production, respectively, and *cyp17*, which also has a role in sex determination (Blázquez et al. 2017; Meng et al. 2019; Narayan 2015; Yang et al. 2021; Zhai et al. 2022). Thus, together these genes are candidate drivers of the fusion, perhaps similar to the emergence of neo‐sex chromosomes in the *Danaini* butterfly (Mora et al. 2024).

While our dataset allowed us to analyse just one heterogamety change to ZW, this change happened on the same chromosome. The two ZW species shared FBGs on the sex chromosome region common to both species, but not on the neo‐sex chromosome part specific to *C. leptosoma* . This indicates an origin of sex bias on LG05 coinciding with its function as ZW sex chromosomes rather than simply being due to species relatedness. We suggest these genes to be involved in the heterogamety change from XY to ZW and as possible candidates under (previous) SAS. These genes consisted of transcription/translation regulating factors and genes regulating or functioning in cell metabolism, cell cycle, meiosis or mitosis and apoptosis, suggesting female‐specific upregulation of these processes following heterogamety change and potentially resolving SAS. There was little overlap of SBGs on LG05 between the XY and ZW species, suggesting that the evolution under an XY system left close to no trace after the transition to ZW.

From our tissue‐specific analysis, we see a clear discrepancy between gene expression in the gonads and the soma. As previously shown in other cichlids (Lichilín et al. 2021), gonads had by far the largest amount of SBGs, dominated by MBGs, in Cyprichromini. This is not surprising given the large structural and functional differences of ovaries and testes. Particularly in the adult specialised tissues, sex‐specific expression networks exist, likely leaving little room for sexual conflict over shared expressed genes (Sánchez‐Baizán et al. 2024; Wilhelm et al. 2025). Correspondingly, we found that sex chromosome and heterogamety type had mostly no effect on SBGs in the gonads. We can thus exclude that the global pattern from our tissue‐combined approach is driven by the gonads.

Across somatic tissues, as in the global model, we detected more FBGs on the ZW sex chromosomes and *vice versa* in the XY system. In addition to the global model, we found somatic FBGs on the sex chromosomes in the ZW species to show an increase in sex bias, especially on the youngest sex chromosome part corresponding to LG13. We did not see the opposite pattern in the XY species. We thus suggest that tissue‐specific sex bias in gene expression evolves differently under the two types of heterogamety, at least in the here‐studied system. Alternatively, the observed pattern of feminisation in sex bias could be a feature of a very young sex chromosome system, and a similar corresponding pattern existed but is no longer visible in the slightly older XY systems we studied. Matching to our observations, more sex‐biased genes were found in species with neo‐sex chromosomes in the genus *Drosophila*, yet no effect of sex chromosomes was visible in gonad gene expression (Minovic and Nozawa 2024). In the Emei Moustache Toad, the sex‐linked homomorphic XY region has both more FBGs and MBGs than autosomes, yet differing from our observation, this difference is only detectable in the gonads, not in the soma (Xie et al. 2025).

Finally, we found more SBGs to be under putative directional selection in *Paracyprichromis* testes and *Cyprichromis* ovaries, and generally more SBGs were under putative directional selection in the females in somatic tissue, suggesting that the gain and loss of SBGs are driven to a greater degree by selection on females. Rapid evolution of female bias has been discovered in some species where females experience stronger selection, such as in farm hens selected for egg‐laying (Moghadam et al. 2012), or in malaria mosquitoes with female‐specific blood‐feeding behaviour (Papa et al. 2017). Species of the genus *Cyprichromis* are maternal mouth brooders with similar mating strategies where males defend three‐dimensional territories to attract mating partners (Konings 2019). They often form large mixed‐species schools. Superficially, they share similar lifestyles and occupy a similar ecological niche (Konings 2019). Still, *C. microlepidotus* typically produces smaller clutches than *C. leptosoma* (Anderson et al. 2016; Konings 2019). Additionally, *C. leptosoma* females often produce clutches of offspring with multiple paternity (Anderson et al. 2016), while field observations of *C. microlepidotus* show that females deposit entire clutches in one male's territory, suggesting single paternity (Ochi 1996). However, multiple paternity cannot be ruled out in *C. microlepidotus* due to the possibility of sneaker males. In conclusion, it is not obvious if selection on males and females differs *per* *se* between XY and ZW species or more generally amongst species.

In summary, we found that a possibly adaptive enrichment of sex bias in gene expression on sex chromosomes can happen very rapidly. Enrichment of SBGs following a heterogamety turnover was primarily driven by the emergence of FBGs in the soma. Moreover, the feminisation in gene expression of LG05 in ZW species was complemented by a demasculinisation of sex‐biased intensity in MBGs. This was coupled with an increase of female‐biased expression and/or a decrease in male‐biased expression depending on the tissue type. Sex chromosomes of the XY species were found to be generally masculinised in the amount of SBGs, though not in the intensity of sex‐biased expression. We demonstrate that the feminisation in ZW‐LG05 and masculinisation of XY‐LG05 and XY‐LG15 is influenced by the heterogamety system in opposing directions and by differing mechanisms. We further show that sex bias in gene expression is different in young sex chromosomes compared to heteromorphic ones. In line with other reports from homomorphic/neo‐sex chromosomes, we propose that the novel sex‐specific expression patterns derive from the sex‐limited chromosome readily after its emergence and thus benefit the heterogametic sex, especially in the female heterogametic species. A sex‐specific evolution of the X/Z chromosomes might simply take longer to evolve.

## Author Contributions

A.B. and K.H. designed the study with input from S.H.S. K.H. carried out data analysis and drafted the manuscript. All authors contributed to writing and data interpretation.

## Disclosure

Benefit‐sharing statement: Benefits from this research might accrue to the general research community from the sharing of our results in this article. Contributions of all individuals to the research are described in the Authors Contributions and Acknowledgements.

## Conflicts of Interest

The authors declare no conflicts of interest.

## Supporting information


**Figure S1:** Distribution of sex‐specific single‐nucleotide polymorphisms.
**Figure S2:** Global gene expression profile per species.
**Figure S3:** Overlap of sex‐biased genes amongst species.
**Figure S4:** Sex bias strength along the genome.
**Figure S5:** Sex‐biased gene expression along the sex chromosomes.
**Figure S6:** Overlap of male‐biased genes (MBGs) and female‐biased genes (FBGs) amongst species for each tissue on autosomes and on LG05.
**Figure S7:** Density plots of Transcript per Million (TPM) expression ratios of all expressed genes in each tissue.
**Figure S8:** Sex‐biased genes in somatic tissues on sex chromosomes.

**Table S1:** Available RNA‐sequencing data for Cyprichromini.
**Table S2:** Sequencing read statistics.
**Table S3:** Datasets of total genes included in differential gene expression analysis.
**Table S4:** List of sex‐biased genes.
**Table S5:** Results of Fisher's exact test to investigate if sex‐biased genes are more likely to be located on sex chromosomes than autosomes.
**Table S6:** Proportion of sex‐biased genes on each chromosome.
**Table S7:** Shared sex‐biased genes on sex chromosomes in *Cyprichromis*.
**Table S8:** Rank–rank hypergeometric overlap (RRHO) analyses of sex‐biased genes.
**Table S9:** Sex‐biased genes (SBGs) under directional selection.
**Table S10:** Results of Fisher's exact test to investigate directional selection of sex‐biased genes in males and females.
**Table S11:** Results of Fisher's exact test to investigate directional selection on sex chromosomes and autosomes.

## Data Availability

Genetic data are derived from a previous study (El Taher, Böhne, et al. 2021) and accessible from the INSDC under BioProject ID PRJNA552202.
