# Supplementary material for: ZW and XY Sex Chromosomes Drive Rapid and Distinctive Evolution of Sex‐Biased Gene Expression
Source: Mol Ecol. 2025 Oct 18;34(22):e70152. doi: 10.1111/mec.70152 (PMC12617055; doi:10.1111/mec.70152)
Supplement: Supplementary file 1 — Figure S1: Distribution of sex‐specific single‐nucleotide polymorphisms. Figure S2: Global gene expression profile per species. Figure S3: Overlap of sex‐biased genes amongst species. Figure S4: Sex bias strength along the genome. Figure S5: Sex‐biased gene expression along the sex chromosomes. Figure S6: Overlap of male‐biased genes (MBGs) and female‐biased genes (FBGs) amongst species for each tissue on autosomes and on LG05. Figure S7: Density plots of Transcript per Million (TPM) expression ratios of all expressed genes in each tissue. Figure S8: Sex‐biased genes in somatic tissues on sex chromosomes. [file MEC-34-e70152-s001.pdf]

MOLECULAR ECOLOGY

Supplemental Information for:

ZW and XY sex chromosomes drive rapid and distinctive evolution of sex-biased gene expression

Kevin Hsiung, Sophie Helen Smith, Astrid Böhne

Table of Contents:

**Figure S1**	Page 2
**Figure S2**	Page 3
**Figure S3**	Page 4
**Figure S4**	Page 5
**Figure S5**	Page 6
**Figure S6**	Page 7
**Figure S7**	Page 8
**Figure S8**	Page 9

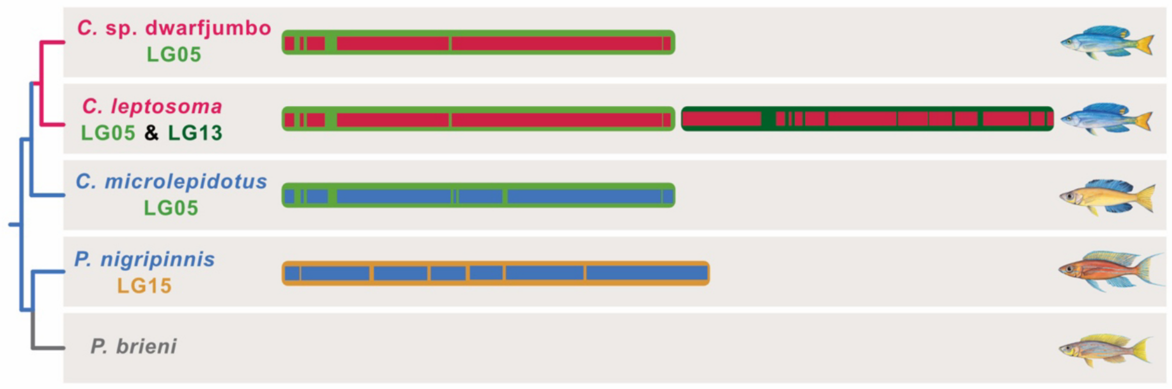

Figure S1. Distribution of sex-specific single nucleotide polymorphisms.
The phylogenetic representation includes the five Cyprichromini species studied for their gene expression profiles. Coloured bars behind species names indicate sex chromosomes. Boxes within bars depict in which region sex-specific SNPs were detected in a previous study (El Taher, Ronco et al. 2021). Colours of bars refer to sex-linked chromosomes in the respective species (light green LG05, dark green LG13, orange LG15). Red colour (species names, branches of phylogenetic tree, boxes for sex-specific SNPs) indicates ZW species, blue color XY species. No sex chromosome has been identified in *P. brieni*.

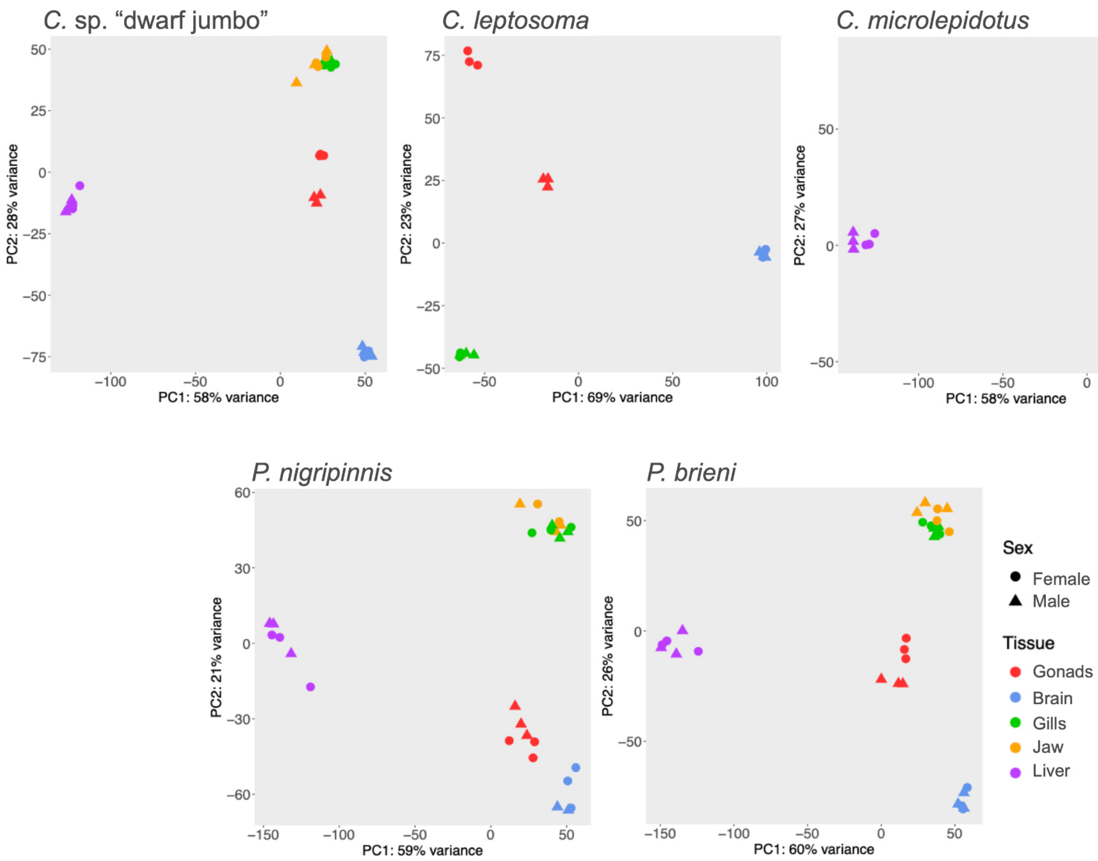

Figure S2. Global gene expression profile per species.
Plots depict principal component analysis of the transcriptomes for each species. Proportions of variance explained by the first two principal components (PC1 and PC2) are indicated in parenthesis on the x- and y-axis, respectively. Samples are coloured according to tissue, and shapes denote sex as depicted in the inset.

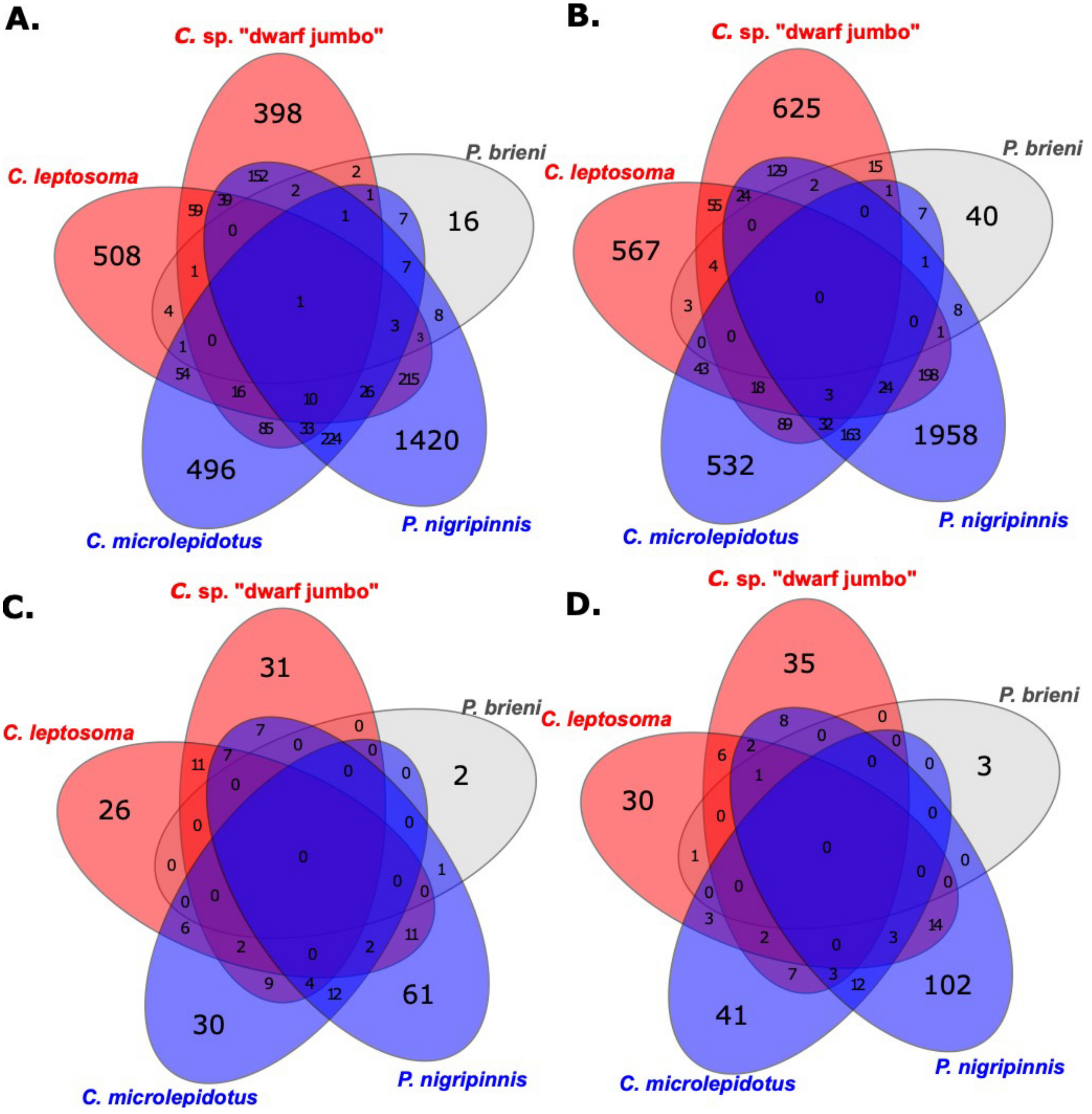

Figure S3. Overlap of sex-biased genes among species.

Venn diagrams show the overlap of MBGs and FBGs between species across all tissues. (A) FBGs on all chromosomes except LG05, (B) MBGs on all chromosomes except LG05, (C) FBGs on LG05, and (D) MBGs on LG05. Species that have ZW sex determination are represented in red, species that have XY sex determination are depicted in blue, *P. brieni* is represented in gray as the heterogametic sex is unknown.

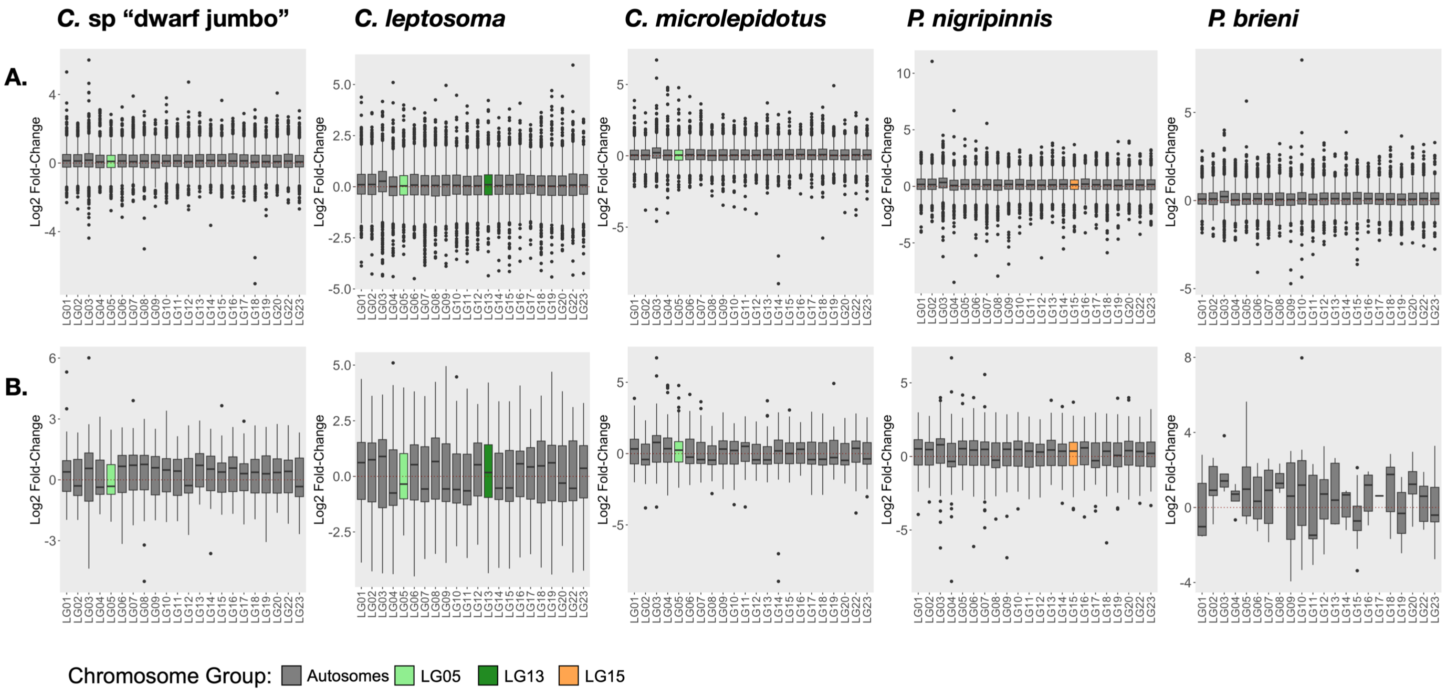

Figure S4. **Sex bias strength along the genome.**

Panels show box plots depicting the male-female log-fold change of expression for all expressed genes on each chromosome. Black line within each box represents the median. The top and bottom boundaries of the box indicate the upper and lower quartiles. Whiskers extend from the hinges to the smallest and largest values within 1.5 times the interquartile range. Sex chromosomes are highlighted with light green for LG05, dark green for LG12 and orange for LG15.

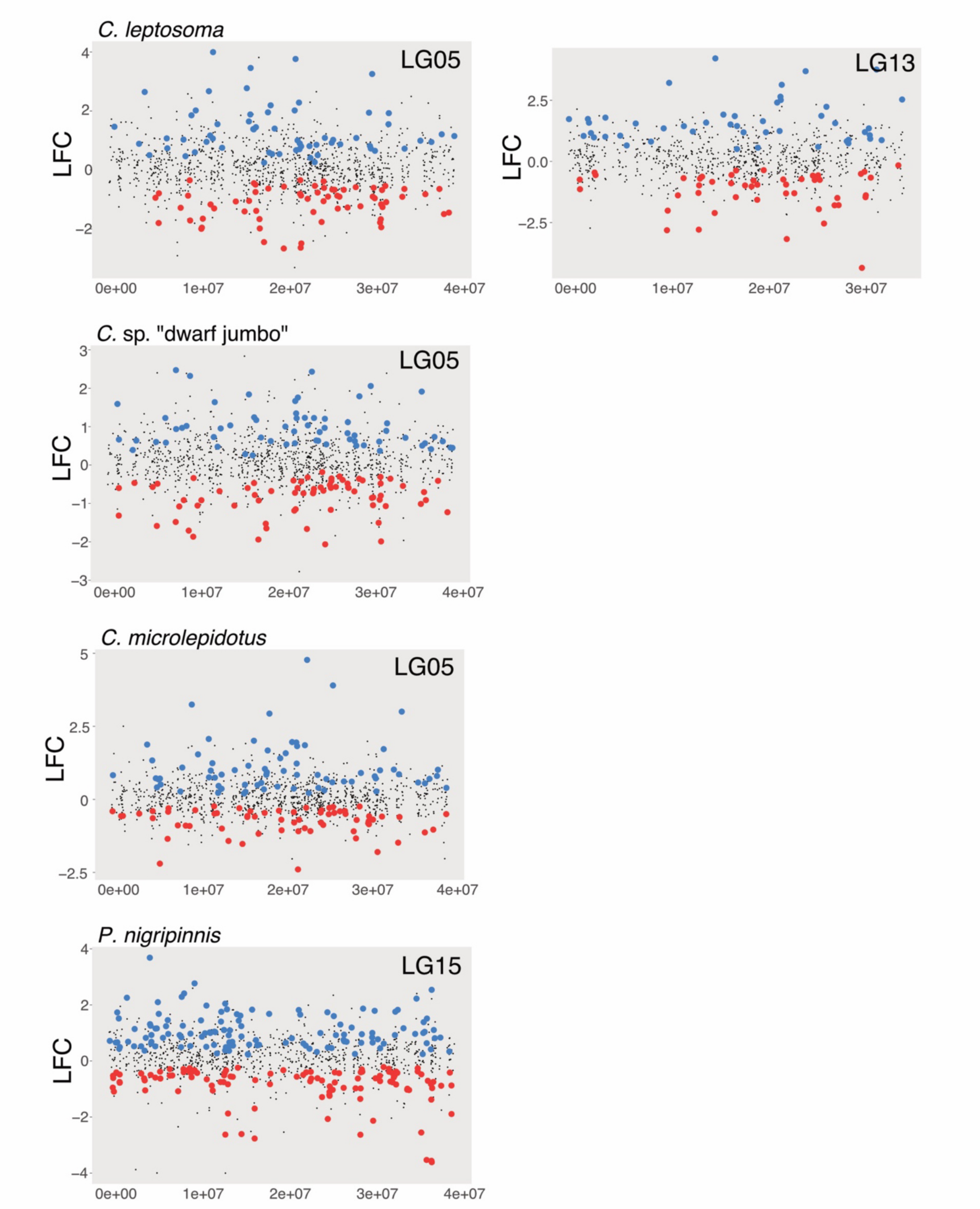

Figure S5. **Sex-biased gene expression along the sex chromosomes.**
Panels show sex-biased expression for each species with male-female log-fold change (LFC) of expression on the y-axis and chromosomal location of the corresponding gene on the x-axis. Red dots indicate significant female-bias, blue indicates male-bias. Black dots are gene with non-significant values.

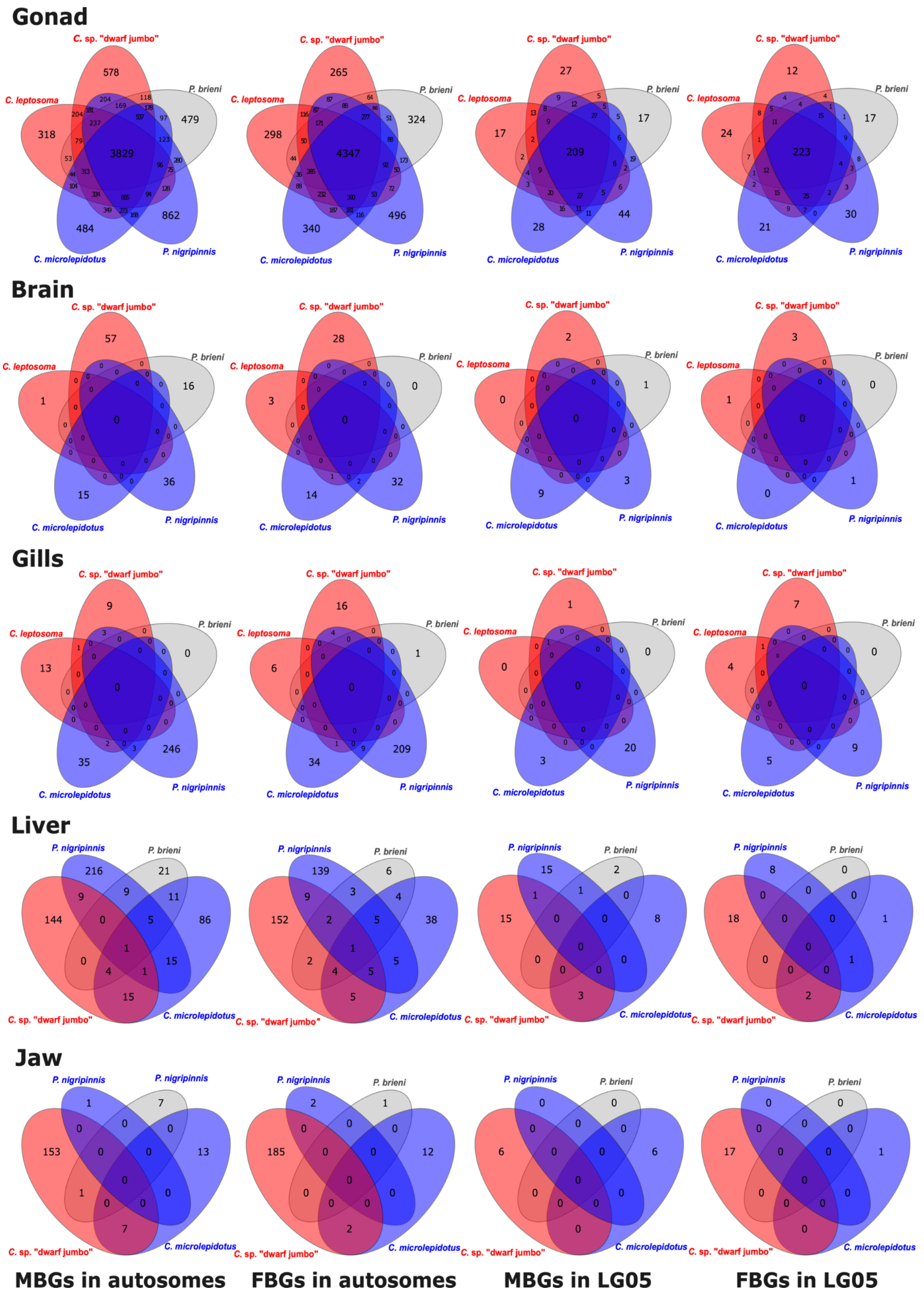

Figure S6. Overlap of male-biased genes (MBGs) and female-biased genes (FBGs) among species for each tissue on autosomes and on LG05.

Species that have ZW sex determination are represented in red, species that have XY sex determination in blue, *P. brieni* is represented in gray as the heterogametic sex is unknown.

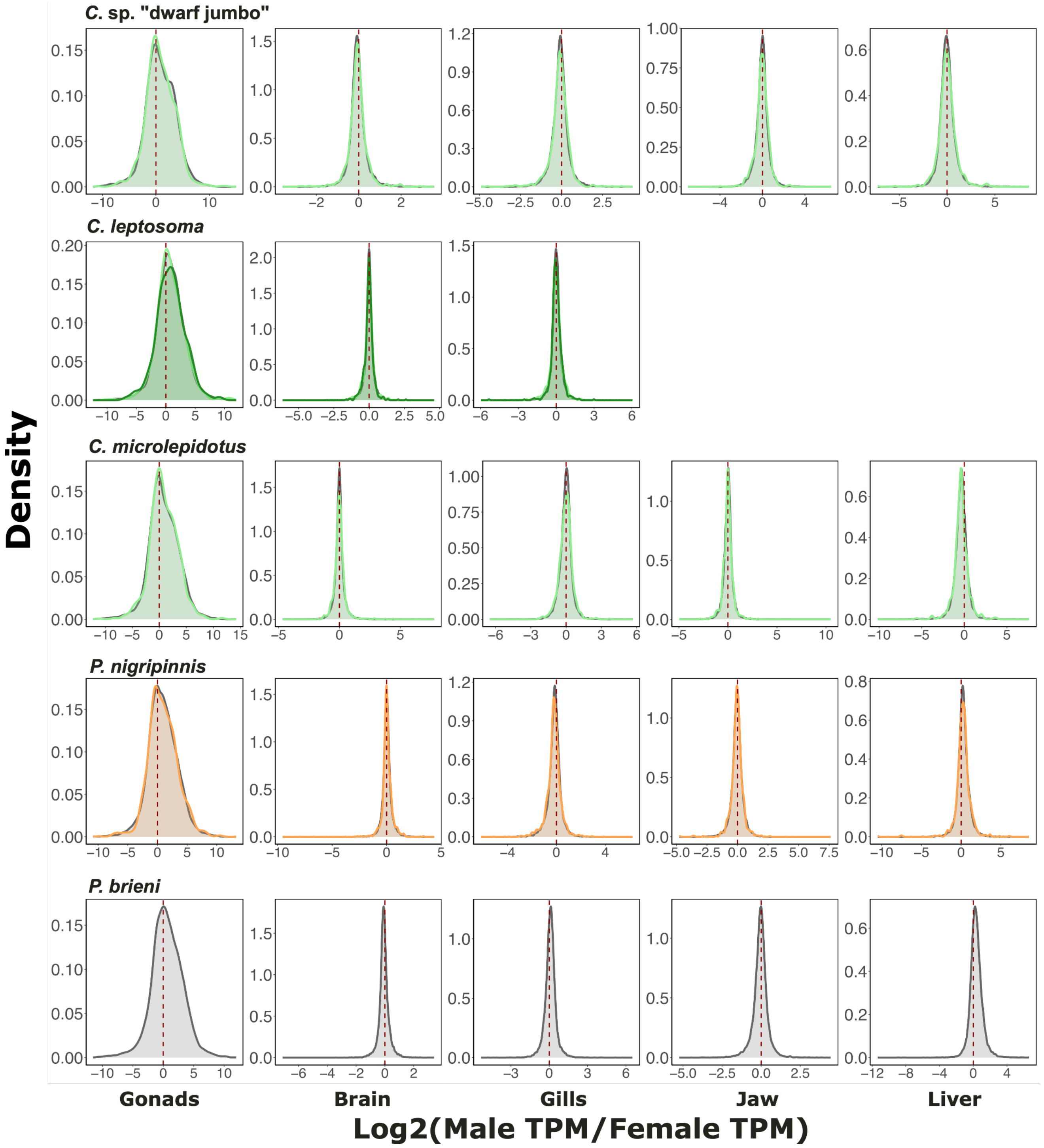

Figure S7. Density plots of Transcript Per Million (TPM) expression ratios of all expressed genes in each tissue.

The density of male to female TPM expression ratios are depicted for each species and tissue. Sex chromosomal and autosomal distributions are superimposed. Gray: autosomes, light green: LG05, dark green: LG13, orange: LG15. Dotted vertical red line: expression ratio of zero.

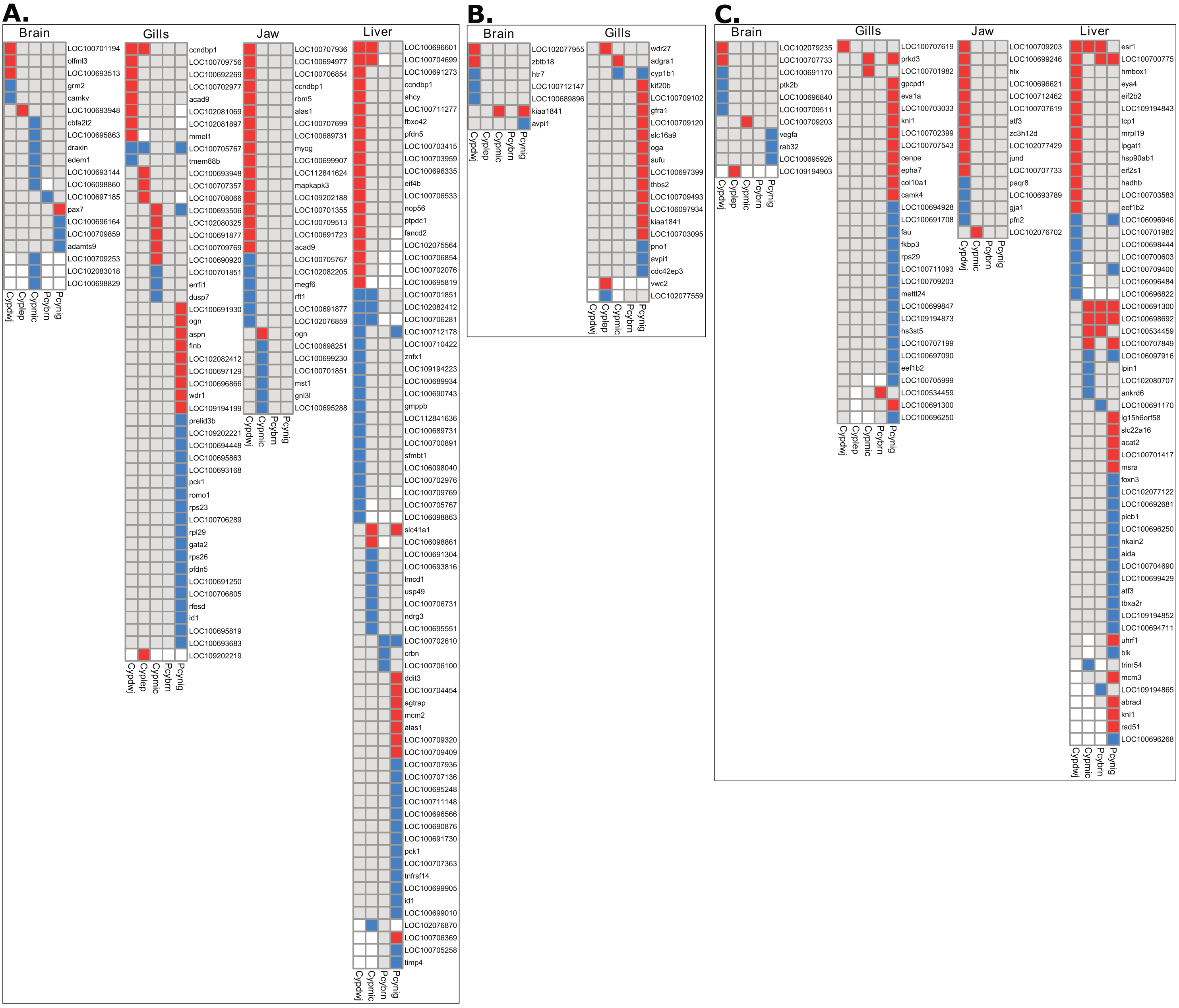

Figure S8. **Sex-biased genes in somatic tissues on sex chromosomes.**

Rows of heatmaps show sex-biased genes (SBGs) of each species with female-biased genes (FBGs) in red, male-biased genes (MBGs) in blue and unbiased genes in grey. White colour indicates that the gene was not expressed. A) LG05, B) LG13, and C) LG15. Species names are abbreviated as follows: Cypdwj = C. sp. “dwarf jumbo”, Cyplep = C. leptosoma, Cypmic = C. microlepidotus, Pcynig = P. nigripinnis, Pcybrn = P. brieni.
